# Increased moral condemnation of accidental harm in institutionalized adolescents

**DOI:** 10.1038/s41598-018-29956-9

**Published:** 2018-08-02

**Authors:** Sandra Baez, Eduar Herrera, Adolfo M. García, David Huepe, Hernando Santamaría-García, Agustín Ibáñez

**Affiliations:** 1 Universidad de los Andes, Bogotá, Colombia; 2 Universidad Icesi, Departamento de Estudios Psicologicos, Cali, Colombia; 3 Laboratory of Experimental Psychology and Neuroscience (LPEN), Institute of Cognitive and Translational Neuroscience (INCYT), INECO Foundation, Favaloro University, Buenos Aires, Argentina; 4 National Scientific and Technical Research Council (CONICET), Buenos Aires, Argentina; 5 Faculty of Education, National University of Cuyo (UNCuyo), Mendoza, Argentina; 6 Center for Social and Cognitive Neuroscience (CSCN), School of Psychology, Universidad Adolfo Ibáñez, Santiago de Chile, Chile; 7 Centro de Memoria y Cognición. Intellectus-Hospital Universitario San Ignacio, Bogotá, Colombia; 8 Physiology, Psychiatry and Aging Institute, Pontificia Universidad Javeriana, Bogotá, Colombia; 9 ACR Centre of Excellence in Cognition and its Disorders, Sydney, Australia; 10 Universidad Autónoma del Caribe, Barranquilla, Colombia

## Abstract

Social deprivation, as faced by children in institutional rearing, involves socio-cognitive deficits that may persist into adolescence. In particular, two relevant domains which prove sensitive to pre-adult neurodevelopment are theory of mind (ToM) and moral judgment (a complex skill which partially depend upon ToM). However, no study has assessed moral evaluation in adolescents with a history of institutional care, let alone its relationship with ToM skills. The present study aims to bridge this gap, focusing on moral evaluation of harmful actions in institutionalized adolescents (IAs). Relative to adolescents raised with their biological families, IAs exhibited less willingness to exculpate protagonists for accidental harms, suggesting an under-reliance on information about a person’s (innocent) intentions. Moreover, such abnormalities in IAs were associated with ToM impairments. Taken together, our findings extend previous findings of delayed ToM under social deprivation, further showing that the development of moral cognition is also vulnerable to the impact of institutionalization. These results could pave the way for novel research on the role of institutional rearing in ToM and moral development during adolescence.

## Introduction

Children in institutional rearing are exposed to social deprivation, which has been associated with important delays in physical, cognitive, behavioral, and social cognition development^1–4^. Specifically, children with a history of institutional care exhibit deficits in basic cognitive skills, such as memory and executive functions (EFs)^1^, as well as in social cognition domains, including emotion recognition^5^, theory of mind (ToM)^2,6,7^, and moral development^3,8^. During adolescence, such impairments persist and may even increase over time^2^.

One social cognitive ability that proves particularly sensitive to neurodevelopment during adolescence is moral evaluation^8,9^. This domain reflects the complex integration of several social-cognitive processes, including ToM^10,11^. However, to date, no studies have assessed multidomain moral evaluation abilities in adolescents with a history of institutional care, let alone their relationship with ToM skills. Against this background, the present study aimed to assess the moral evaluation of harmful actions in institutionalized adolescents (IAs) and a control group of adolescents raised with their biological families. We also explored the relationship between moral evaluation and ToM abilities in IAs.

Socially deprived adolescents have deficits in their moral development^8^ and show atypical brain activity underlying performance in moral sensitivity’s tasks^3^. Indeed, abnormal moral development in maltreated adolescents has been associated with increased aggression and higher rates of delinquency^12^. Conceivably, such patterns may be linked to alterations in underlying domains, such as ToM^10,11^. In neurotypicals, moral judgment of an action is usually determined primarily by the agent’s intention rather than the outcome^13^. However, when intention and outcome are in conflict, moral judgments rely on the consideration of the agent’s mental state and the harmfulness of his doing^11,14,15^. Thus, when judging an action producing accidental harm (e.g., unintentionally hitting someone), individuals must weigh in the agent’s intention to override a preponderant negative response to the outcome^15^. Of note, this ability relies heavily on ToM^10^.

Maturation of such a domain proves delayed in institutionalized children^2,6,7^. Compatibly, relative to non-maltreated children with low socioeconomic status, those who experience maltreatment are at risk for delayed ToM development^7,16^. These socio-emotional difficulties seem to persist or even increase over time in some children^7^. Thus, considering the relationship between moral judgment and ToM, and the reported difficulties in the latter domain, ToM impairments could be associated with moral judgment abilities in IAs.

Although some research has found moral cognition impairments in socially deprived adolescents^3,8^, no study has specifically assessed the moral judgment of other’s harmful actions in IAs, let alone its relationship with ToM abilities. This study aimed to bridge such gaps comparing IAs with matched adolescents raised with their biological families. Two relevant tasks were used to this end. First, we employed a well-characterized moral judgment task^17–20^ involving scenarios that disentangle the contributions of intentions and outcomes to moral evaluation. Second, we used an experimental paradigm^21–25^ assessing the perception and evaluation of harm in the context of intentional and accidental actions. In addition, we explored the relationship between moral evaluation and ToM abilities in IAs. Finally, we explored whether moral evaluation judgments were associated with relevant cognitive factors, such as EFs. We hypothesized that IAs would exhibit ToM impairments and abnormal moral evaluation for situations that require considering a perpetrator’s mental state to override a preponderant negative response to the outcome (i.e., accidental harm). Moreover, we expected this moral evaluation pattern to be associated with ToM impairments. In short, through this study, we expect to open new avenues on the role of institutional rearing in the development of moral cognition and ToM during adolescence.

## Results

### Intellectual level and EFs

We assessed fluid intelligence given its possible impact on ToM and moral evaluation performance. Results showed no significant differences between groups in fluid intelligence. However, IAs showed a significantly lower executive functioning than controls (see Table 1).

**Table 1:** Demographic data, intellectual and executive functions.

		IAs (*N* = 35)	Controls (*N* = 22)	*p* values
		Mean [95%CI]	Mean [95%CI]	
**Demographics**				
	Age (years)	15.68 [14.95, 16.42]	16.0 [15.14, 16.85]	0.57
	Gender (F:M)	0:35	0:22	1.00
	Education (years)	8.65 [7.78, 9.63]	9.40 [8.48, 10.33]	0.25
**Fluid intelligence and executive functions**				
	Raven’s standard progressive matrices	33.60 [32.10, 35.09]	32.63 [29.24, 36.03]	0.54
	IFS total score	18.77 [17.76, 19.78]	21.40 [20.37, 22.43]	<0.001
IFS subscales	Motor series	2.80 [2.59, 3.00]	2.68 [2.47, 2.89]	0.42
	Conflicting instructions	2.80 [2.66, 2.93]	2.81 [2.64, 2.99]	0.86
	Go- no go	2.80 [2.63, 2.96]	2.86 [2.70, 3.00]	0.58
	Backward digits span	3.11 [2.79, 3.43]	2.86[2.31, 3.41]	0.38
	Verbal working memory	1.22 [0.96, 1.49]	1.68 [1.39, 1.96]	<0.05
	Spatial working memory	1.85 [1.57, 2.13]	2.68 [2.30, 3.05]	<0.001
	Abstraction capacity	0.71 [0.41, 1.00]	1.45 [1.05, 1.85]	<0.01
	Verbal inhibitory control	3.45 [2.90, 4.01]	4.36 [3.41, 4.20]	<0.05

IFS: INECO Frontal Screening battery.

### Task A: Moral judgment

Participants performed a well-characterized moral judgment task^14,15^ (see Methods section) that disentangles the contributions of intentions and outcomes to moral judgment. The task included two conditions in which intentions and outcomes matched (i.e., “no harm intended or inflicted” and “intentional harm”) and two in which these variables mismatched (i.e., “unsuccessfully attempted harm” and “accidental harm”).

Actions with neutral intentions and neutral outcomes were judged as more permissible than actions with negative intentions and negative outcomes [main effects of intention (*F*(1, 54) = 173.99, *p* < 0.001, η^2^ = 0.76) and outcome (*F*(1, 54) = 102.58, *p* < 0.001, η^2^ = 0.65)]. Furthermore, accidental harm was judged as more permissible than intentional harm [intention x outcome interaction (*F*(1, 54) = 24.48, *p* < 0.001, η^2^ = 0.31)].

We also observed an interaction between intention and group (*F*(1, 54) = 18.20, *p* < 0.001, η^2^ = 0.25). Planned comparisons revealed that IAs judged accidental harm as less permissible than did the controls (*F*(1, 54) = 16.80, *p* < 0.001, η^2^ = 0.24). This group difference remained significant after adjusting for EFs (*F*(1, 53) = 7.94, *p* < 0.01, η^2^ = 0.13). A significant effect of EFs on accidental harm judgments was also observed (*F*(1, 53) = 4.30, *p* < 0.05, η^2^ = 0.07).

**Figure 1:**
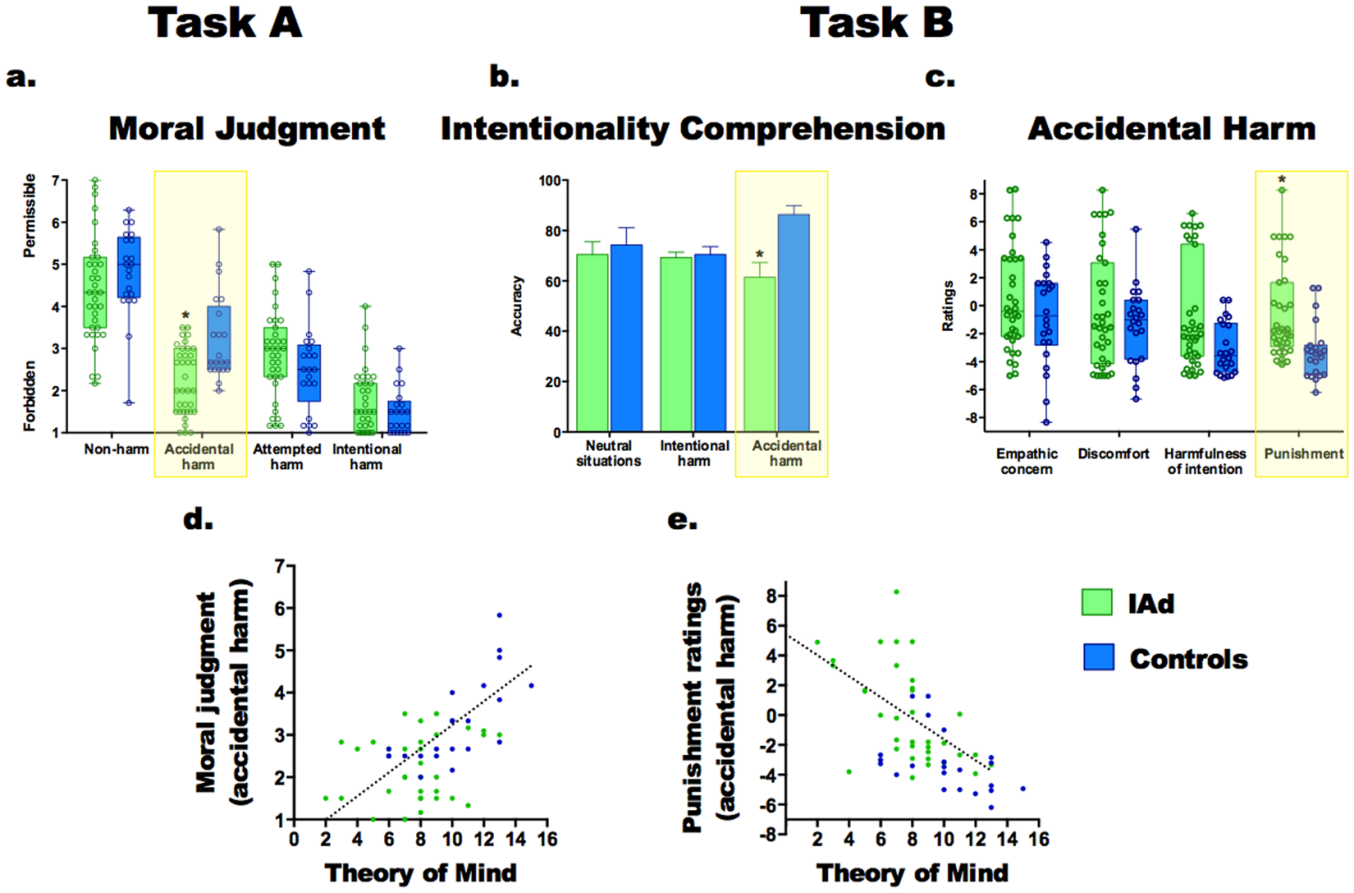
Significant differences between groups and associations between moral evaluation and ToM. (**a**) Moral judgments of IAs and controls. (**b**) Intentionality comprehension accuracies of IAs and controls. (**c**) Data from Task B, ratings for accidental harms. Asterisks indicate significant differences (*p* < 0.05). (**d**) Regression analysis with moral judgment of accidental harms as dependent variable and ToM score as significant predictor. (**c**) Regression analysis with punishment ratings for accidental harms as dependent variable and ToM score as significant predictor.

No significant differences were observed for situations marked by non-harm (*F*(1, 54) = 1.55, *p* = 0.21, η^2^ = 0.02), attempted harm (*F*(1, 54) = 1.57, *p* = 0.21, η^2^ = 0.02) or intentional harm (*F*(1, 54) = 1.18, p = 0.28, η^2^ = 0.02) (Fig. 1a).

Intra-group comparisons revealed that IAs judged accidental harm as less permissible than attempted harm. The opposite pattern was observed in controls (see S4).

### Task B: Intentionality comprehension and moral evaluation task

Participants also completed an intentionality comprehension and moral evaluation task, which includes five different measures (see methods section). Regarding intentionality comprehension, a significant interaction emerged between group and condition (*F*(2, 110) = 3.66, *p* < 0.05, η^2^ = 0.07). A post-hoc analysis (Tukey’s HSD, *MS* = 655.51, *df* = 164.59) revealed that, compared to controls, IAs had lower scores in intentionality comprehension (*p* < 0.001) (Fig. 1b), evidencing difficulties in the ability to distinguish accidental situations from intentional ones. This difference did not remain significant after adjusting for EFs (*F*(1, 53) = 2.16, *p* = 0.14, η^2^ = 0.03).

Furthermore, a significant interaction between group and condition was observed in ratings of harmfulness of intention (*F*(2, 110) = 3.29, *p* < 0.05, η^2^ = 0.09). A post-hoc analysis (Tukey’s HSD, *MS* = 10.79, *df* = 162.57) showed that IAs tend to show higher ratings than controls for accidental harm (*p* = 0.08). This tendency was preserved after co-varying by EFs (*F*(1, 53) = 3.84, *p* = 0.06, η^2^ = 0.09).

Punishment ratings were also characterized by a significant interaction between group and condition (*F*(2, 110) = 12.50, *p* < 0.001, η^2^ = 0.12). A post-hoc analysis (Tukey’s HSD, *MS* = 9.61, *df* = 150.62) showed that IAs rated accidental harm higher than controls (*p* < 0.001) –Fig. 1c. This group difference remained significant after adjusting for EFs (*F*(1, 53) = 8.65, *p* < 0.01, η^2^ = 0.13).

No significant interactions between group and condition were observed for ratings of empathic concern (*F*(2, 110) = 2.06, *p* = 0.13, η^2^ = 0.003) or discomfort (*F*(2, 110) = 1.10, *p* = 0.33, η^2^ = 0.01). No reaction-time differences were observed between groups.

### ToM

IAs (*M* = 7.77, *SD* = 2.54) showed a lower performance than controls (*M* = 10.13, *SD* = 2.60) in the Reading-the-Mind-in-the-Eyes Test (RMET) (*F*(1, 54) = 11.45, *p* < 0.01, η^2^ = 0.17). This effect remained significant after adjusting for EFs (*F*(1, 53) = 4.30, *p* < 0.05, η^2^ = 0.07). However, a significant effect of EFs on ToM was also observed (*F*(1, 53) = 11.82, *p* < 0.01, η^2^ = 0.18)”.

### The relationship between moral evaluation, EFs and ToM

We conducted multiple regression analyses to explore the association between ToM, EFs, and those moral evaluation measures yielding significant between-group differences (see Data Analysis). For these models, variance inflation factors (VIF) analyses revealed no evidence of multicollinearity between predictors (see Table 2). A first model including moral evaluation for accidental harm (Task A) as dependent variable (*F*(3, 53) = 12.00, *p* < 0.001, R^2^ = 0.40) revealed that group (beta = −0.27, η^2^ = 0.08) and ToM (beta = 0.41, η^2^ = 0.16) were significant predictors (Fig. 1d). A second model including intentionality comprehension of accidental harm (Task B) as dependent variable (*F*(3, 53) = 9.48, *p* < 0.001, R^2^ = 0.31) showed that EFs (beta = 0.48, η^2^ = 0.19) was the only significant predictor associated with the dependent variable. We carried out a third model with punishment for accidental harm (Task B) as dependent variable. This model (*F*(3, 53) = 13.59, *p* < 0.001, R^2^ = 0.40) evidenced that group (beta = 0.26, η^2^ = 0.08) and ToM (beta = −0.46, η^2^ = 0.20) were significantly associated with punishment ratings (Fig. 1e).

**Table 2:** Variance inflation factors for predictors included in multiple regression analyses.

Independent factors	VIF
EFs	1.5
ToM	1.473
Group	1.309

## Discussion

This is the first study investigating moral judgment and its relationship with ToM in IAs. Convergent evidence from two tasks showed that these subjects exhibit a specific moral evaluation pattern characterized by less willingness to exculpate protagonists for accidental harm. In judging such scenarios, IAs seem to attach little importance to a person’s innocent intentions, thus over-relying on the action’s negative outcome. Moreover, IAs evidenced ToM impairments, which, in turn, were associated with the abnormal moral evaluation of accidental harm. These results could pave the way for novel research on the role of institutional rearing in ToM and moral development during adolescence.

### Moral evaluation and ToM

Results of the moral judgment task (Task A) showed that IAs were less willing than controls to exculpate protagonists for accidental harm, inflicted despite innocent intentions. Moreover, whereas the control group judged accidental harm as less morally wrong than attempted harm, IAs showed the opposite pattern. Thus, in judging accidental harm, IAs seemed to rely less heavily on information about innocent intentions, thus attaching greater importance to negative outcomes in themselves. Of note, judging the morality of an action based on the analysis of a person’s intentions requires ToM skills^10,15^. The IAs group exhibited ToM impairments. Thus, abnormal moral judgment of accidental harm in IAs could be influenced by their ToM deficits.

The moral judgment pattern observed in IAs resembles that observed at early developmental stages^26,27^. By age 4, children are likely to assign more moral weight to outcomes than intentions when evaluating actions^26^. The proclivity to use belief information to exculpate people for accidental harms increases in neurotypical children from ages 5 to 11^28^. Although all participants in the IA group were above age 12, they exhibited an immature pattern of moral judgment. This finding is consistent with previous research revealing that socially deprived adolescents have deficits in their moral development^8^.

Similar results were found in the assessments of intentionality comprehension and moral evaluation (Task B). Regarding intentionality comprehension, compared to controls, IAs misinterpreted accidental harm as intentional. This ability to distinguish accidental from intentional harm requires inferring the intentionality behind others’ actions. Interestingly, better comprehension of intentionality for intentional than accidental harm has been previously reported in non-clinical populations^9^. This is presently observed for IAs but not for controls, who showed a higher accuracy for accidental harm than for neutral situations and intentional harm. These differences between studies probably reflect demographic characteristics of the samples (i.e., age, educational level or cognitive functioning) and methodological discrepancies (i.e., the paradigm employed). Indeed, several previous studies^22,23,25,29^ using the task employed here showed that, in control subjects, intentionality comprehension for accidental harm is similar or even higher than for intentional harm. Future research should explore these sources of variability and further investigate the factors associated with intentionality comprehension in institutionalized and other adolescent populations.

The detection of intentionality is a decisive step to determine whether an action was malicious^9,30^. The inability to carry out such a process may affect ratings of harmfulness of intention and punishment. In this sense, IAs tended to give higher harmfulness of intention ratings and assigned significantly more punishment to accidental situations than controls. However, accidental harm may go unpunished, regardless of its magnitude^15,31^. Thus, once again, these findings could suggest deficits in inferring the intentionality of others’ actions.

Together, our results are consistent with those of previous research showing basic moral sensitivity impairments in socially deprived adolescents^3,8^. These findings extend the understanding of such deficits by providing evidence of a specific abnormal pattern in the moral evaluation of accidental harm. Considering that impaired moral development in maltreated adolescents has been associated with increased aggression and higher rates of delinquency^12^, future studies should assess whether the moral evaluation pattern observed here is related to behavioral deficits in IAs.

Of note, the moral evaluation abnormalities observed in IAs across both tasks were restricted to judging accidental harm. In Task A, no between-group differences emerged in any other kind of scenarios. The same was true for intentional harm and neutral situations ratings in Task B. This specific pattern of enhanced moral condemnation of accidental harm suggests that IAs are less willing to exculpate protagonists for accidental harm caused on the basis of innocent intentions. Exculpation for accidental harm requires a robust construal of the agent’s mental state to override a proponent response to the salient information about actual harm^11,15^. This aligns with the view that abnormal moral evaluation of accidental harm in IAs might be related to reduced ToM skills.

Supporting the specificity of this moral evaluation pattern, no between-group differences were observed in affective aspects of action evaluation as assessed by Task B, namely: discomfort and empathic concern. Both, these variables point to affective domains closely linked with empathic abilities^32^. The degree of discomfort involves self-oriented feelings of personal unease when exposed to the suffering of others. While discomfort may produce a motivation to reduce one’s own personal distress, empathic concern may instigate an altruistic motivation to help others. Thus, these are self- and other-oriented empathic responses that are not associated with ToM abilities^9^. Their preservation in IAs reinforces the specificity of their moral judgment pattern and the latter’s potential grounding in ToM.

Previous reports have suggested that increased moral judgments in the face of accidental harms may be explained either by ToM deficits^33^ or elevated sensitivity in perceiving the victim’s experience of pain^34–36^. However, as implied by our results, a more plausible explanation for the moral evaluation pattern observed in IAs is that these participants possess reduced ToM skills, affecting their ability to assess the agents’ innocent intentions in accidental-harm scenarios. Further research should study the specific relationship between moral judgment and empathic abilities in IAs.

ToM deficits observed in IAs are consistent with previous studies^2,6,7^ showing that children in institutional rearing show delayed maturation of ToM. Moreover, our results support the observation^7^ that such impairments may persist over the adolescence. Tentatively, this pattern might be partially influenced by parental (physical or psychological) abuse received by some of the IAs in our study. Indeed, relative to non-maltreated children with low socioeconomic status, those who experience maltreatment are at risk for delayed maturation of ToM^7,16^. Future studies should specifically assess the relationship between ToM impairments and the history of parental abuse.

Our ANCOVA results showed that EFs had a significant effect on ToM. This finding aligns with previous evidence of a relationship between EFs and ToM^37,38^. ToM capacity requires the active inhibition of one’s own perspective to understand other’s mental states. Thus, EF deficits observed in IAs may affect both ToM ability as well as the capacity to evaluate accidental harm properly. The specific contribution of EFs to ToM and moral judgment in IAs should be assessed in future research.

Besides, further studies should employ additional measures of ToM considering both affective and cognitive mental state inference. Note that the ToM measure used in this study has been argued not to be an optimal measure of this construct. The RMET includes emotional states and relies on the detection of subtle facial cues, features typically used to test emotion recognition^39^; however, the comparison between this test and emotion tasks employing visual facial stimuli^40^ and verbal auditory stimuli^41^ reveals a clear distinction: both emotion tasks are tackling basic emotions^42^, while the RMET involves additional ToM components^43,44^. Notwithstanding, future research in IAs should include other ToM tasks involving the inference of non-emotional mental states.

### The relationship between moral evaluation, EFs, and ToM

Moral judgment for accidental harm and punishment ratings for accidental harm were significantly associated with ToM abilities. These findings support the assumption that a delay in ToM development observed in IAs influences moral evaluation abilities. As mentioned above, atypical moral judgment was restricted to the evaluation of accidental harm scenarios, which crucially rely on the consideration of a perpetrator’s mental state to override a preponderant negative response to the outcome. Thus, this selective pattern characterized by increased moral condemnation of accidental harm could be explained by ToM deficits in IAs.

Moreover, in line with a previous study^45^, ANCOVA results showed that EFs may contribute to abnormalities in the moral evaluation of accidental harm. Specifically, inhibitory control resources enabling regulation and control of other cognitive processes might be particularly critical for judging accidental harm^45^. Although ToM and moral results in IAs might be influenced by EFs, collinearity analyses revealed that results are not fully explained by outcomes in such a domain. This pattern of results aligns with previous studies in different populations showing a relationship, but no collinearity, between EFs and social cognitive measures^3,24,46^. To gain insights on this issue, further research should study the specific relationship between EFs, ToM and moral judgment in IAs.

In addition, comprehension of the intentionality behind accidental harm was predicted by EFs, but not by ToM skills. Consistent this finding, ANCOVA results showed that group differences in intentionality comprehension of accidental harm disappeared after adjusting for EFs. Together, these results suggest that accurate recognition of these situations may involve preserved executive functioning. This is consistent with the results of previous studies in clinical populations^21,23^ showing that deficits in intentionality comprehension of accidental harm are associated with executive dysfunction. However, the specific links between EFs and moral cognition in IAs requires further assessments through more comprehensive tools.

### Conclusion

IAs exhibited a specific moral evaluation pattern characterized by less willingness to exculpate protagonists for accidental harm and a tendency to assign more punishment to them. This suggests an underreliance on information about a person’s (innocent) intention, accompanied by an overreliance on the action’s negative outcome. Moreover, IAs exhibited ToM deficits, suggesting that similar impairments reported in children under institutional rearing can persist into adolescence. In addition, such ToM impairments accounted for the subjects’ deviant judgment of accidental harm. Taken together, our findings extend previous findings of delayed ToM under social deprivation, further showing that the development of moral cognition is also vulnerable to the impact of institutionalization. A subtler understanding of these sociocognitive abnormalities in IAs may shed light on potential strategies for early for early detection of potentially dangerous profiles and for the development of stimulation programs. Finally, our results offer new insights into the neurodevelopment of ToM and morality after social deprivation, thus opening promising avenues for further research.

## Methods

### Participants

Our sample included 35 male IAs (aged between 12 and 19) and a control group of 22 male adolescents raised with their biological families. IAs had been institutionalized in a center located in Cali (Colombia), at a mean age of 10.51 (*SD* = 2.22, range 3–13) and remained so for a range of 1 to 13 years (*M* = 5.17, *SD* = 3.18). Twelve of these adolescents had parents who could not provide adequate care and 23 had no knowledge of the location of their biological mother or father. All adolescents were institutionalized due to extreme poverty conditions (i.e., inability to satisfy basic needs in food, clothing, shelter, and health), neglect, and/or physical or psychological abuse from their parents or tutors. All IAs and controls were regularly attending school at the time of testing. They possessed normal weight and size, and they were affiliated to a health care system.

We controlled for between group differences in age, sex, and education level (Table 1). Participants had no history of drug and/or alcohol abuse, psychiatric or neurological disorders, as assessed through an interview with the parents and the institutions’ records. Participants with history of developmental or behavioral disorders were excluded. Also, IAs and controls resided in the same district, which minimized gross cultural differences between samples. Adolescents and parents gave informed consent in agreement with the Declaration of Helsinki. All experimental procedures were approved by the Ethics Committee of ICESI University. All procedures in this study were conducted in accordance with the relevant guidelines and regulations of the Declaration of Helsinki.

### Instruments

#### Intellectual level and EFs

Fluid intelligence was assessed via Raven’s standard progressive matrices^47^ (the maximum score on this test is 60 points). EFs were evaluated through the INECO frontal screening (IFS) battery^48^, a sensitive tool to detect executive dysfunction^48,49^ (for details, see S1).

#### Moral evaluation tasks

Task A: Moral judgment task: Following a previously reported protocol^17,20,50^, we presented the participants with 24 scenarios. Four variations of each scenario were included, following a 2 × 2 design: (1) the protagonists either harmed another person (negative outcome) or induced no harm at all (neutral outcome); (2) the protagonists either believed that they would cause harm (negative intention) or believed that they would cause no harm (neutral intention). Each possible belief was true for one outcome and false for the other. Thus, the four scenarios were: (1) no harm, (2) accidental harm, (3) attempted harm, and (4) intentional harm. After reading each story, the participants were asked to rate the moral nature of each scenario on a seven-point Likert scale ranging from totally forbidden (1) to totally permissible (7) (for details, see S2).

Task B: Intentionality comprehension and moral evaluation task: This task evaluates different aspects of intentionality comprehension, empathy, and moral evaluation in the context of intentional and accidental harms. Stimuli consisted in 11 animated scenarios (4 intentional, 4 accidental, 3 neutral) featuring two individuals. This task has been previously employed in several studies^21–25,29,30,50^. In all scenarios, motion was implied by the successive presentation of three digital color pictures. After viewing each sequence, participants responded to five questions evaluating: (a) comprehension of the agent’s intention (was the action done on purpose?), (b) empathic concern (how sad do you feel for the victim?), (c) degree of discomfort (how upset do you feel for what happened in the situation?), (d) harmfulness of intention (how bad was the agent’s intention?), and (e) punishment (how much penalty does this action deserve?). The question about intentionality was answered by selecting “Yes” or “No”. The other questions were answered using a computer-based visual analogue scale ranging from −9 to 9 (these numbers were not visible to participants). The meaning of the scale’s extreme values depends on the question. For example, in the question “how sad do you feel for the hurt person?”, one extreme of the bar reads “I feel very sad” and the other extreme reads “I don’t feel sad at all” (for details, see S3).

#### ToM

ToM was assessed with the RMET. This instrument has been successfully employed to assess ToM in skills in adults who retrospectively reported experiences of parental maltreatment in childhood^51^. The RMET evaluates emotional inference dimensions of ToM^52^. This is a computerized and validated test which consist of 17 pictures of the eye region of a face. Participants are asked to choose which of four words best describes what the person in each photograph is thinking or feeling.

### Data analysis

The assumption of normality was verified using the Shapiro-Wilk test. Data also met the assumption of homogeneity of variance, assessed with Levene’s test. Demographic, neuropsychological, and experimental data were compared between groups with ANOVA and Tukey’s HSD post-hoc tests (when appropriate). Following the procedure reported elsewhere^18,20,53^, data from Task A was analyzed via a 2 (intention: neutral, negative) × 2 (outcome: neutral, negative) × 2 (group: IAs, controls) repeated-measures ANOVA. Paired-sample *t*-tests were used to compare intra-group performance on the moral conditions in which IAs differed from controls. For Task B, ratings for each question were analyzed independently using a 2 (group: IAs vs. controls) × 3 (condition: intentional, accidental, neutral) factorial ANOVA. Following previous procedures^21–23,25,29^, when a significant interaction between group and condition was found, we examined between-group differences using the Tukey’s HSD post-hoc test. Also, in light of the previously established relationship between EFs and moral judgment^45^, on the one hand, and ToM^37,38^, on the other, we re-analyzed our data using the total IFS score as a covariate.

In addition, we conducted multiple regression analyses to explore the association between ToM, EFs, and those moral evaluation measures yielding significant between-group differences. We estimated three different models in which moral evaluation (Task A), intentionality comprehension, and punishment ratings for accidental harm (Task B) were respectively considered as dependent variables. The following variables were included as predictors: group, IFS, and RMET total scores. For multiple regression analyses, multicollinearity of independent variables was assessed using VIF with a reference value of three before interpreting the final output^54^. We also calculated the correlation matrix between variables for the IA group (see Supplementary Table 1).

Effect sizes were calculated through partial eta (η^2^) tests. The statistical significance level was set at *p* < 0.05.

### Data availability

The data that support the findings of this study are available from the corresponding author upon reasonable request.

## Electronic supplementary material

Supplementary Information
