# Supplementary material for: Increased moral condemnation of accidental harm in institutionalized adolescents
Source: Sci Rep. 2018 Aug 2;8:11609. doi: 10.1038/s41598-018-29956-9 (PMC6072742; doi:10.1038/s41598-018-29956-9)
Supplement: Supplementary file 1 — Supplementary Information [file 41598_2018_29956_MOESM1_ESM.doc]

**Increased moral condemnation of accidental harm in institutionalized adolescents**

Sandra Baez ^a,#^, Eduar Herrera ^b,#^, Adolfo M. García ^c,d,e^, David Huepe ^f^,

Hernando Santamaría-García ^g,h^, Agustín Ibáñez ^c,d,f,i,j,*^

^a^ Universidad de los Andes, Bogotá, Colombia

^b^ Departamento de Estudios Psicológicos, Universidad ICESI, Cali, Colombia

^c^ Laboratory of Experimental Psychology and Neuroscience (LPEN), Institute of Cognitive and Translational Neuroscience (INCYT), INECO Foundation, Favaloro University, Buenos Aires, Argentina

^d^ National Scientific and Technical Research Council (CONICET), Buenos Aires, Argentina

^e^ Faculty of Education, National University of Cuyo (UNCuyo), Mendoza, Argentina

^f^ Center for Social and Cognitive Neuroscience (CSCN), School of Psychology, Universidad Adolfo Ibáñez, Santiago de Chile, Chile

^g^ Centro de Memoria y Cognición. Intellectus-Hospital Universitario San Ignacio, Bogotá Colombia.

^h^ Physiology, Psychiatry and Aging Institute, Pontificia Universidad Javeriana, Bogotá, Colombia.

^i^ ACR Centre of Excellence in Cognition and its Disorders, Sydney, Australia

^j^ Universidad Autónoma del Caribe, Barranquilla, Colombia.

^#^ Equal contribution

* Corresponding author: Agustín Ibáñez, PhD, Laboratory of Experimental Psychology and Neuroscience (LPEN), Institute of Cognitive and Translational Neuroscience (INCYT), Pacheco de Melo 1860, Buenos Aires, Argentina, Postal Code 1126. Phone/Fax: +54 (11) 4807-4748. aibanez@ineco.org.ar

**Supplementary Material**

**S1. Assessment of executive functions**

Executive functions (EFs) were evaluated through the INECO frontal screening (IFS) battery . This battery includes eight subtests: (a) motor programming (Luria series, “fist, edge, palm”); (b) inhibition (subjects are asked to hit the table once when the administrator hits it twice, or vice versa); (c) motor inhibitory control; (d) numerical working memory (backward digit span); (e) verbal working memory (months backwards); (f) spatial working memory (modified Corsi tapping test); (g) abstraction capacity (inferring the meaning of proverbs); and (h) verbal inhibitory control (modified Hayling test). The maximum global score on the IFS is 30 points.

**S2. Moral judgment task**

In total, eight possible versions of the 24 scenarios were presented, with six trials of each of the four conditions. The stimuli were administered in pseudorandom order and the conditions were counterbalanced across participants. After reading each story, the participants were asked to rate the scenario on a Likert scale ranging from totally forbidden (1) to totally permissible (7). To reduce working memory load, the entire text remained visible for the duration of the trial.

**S3. Purpose comprehension and moral evaluation task**

In each scenario, motion was implied by the successive presentation of three digital color pictures. The durations of the first, second, and third pictures in each animation were 500, 200, and 1000 ms, respectively. In the intentional harm scenarios, one person deliberately inflicted pain on another (e.g., by purposely stepping on his/her toe). In the accidental harm scenarios, one person accidentally inflicted pain on another (e.g., by hitting him/her with a bat). In the neutral scenarios, both persons interacted in the absence of pain (e.g., by exchanging flowers). Importantly, since the protagonists’ faces were not visible, facial emotional reactions were factored out from the task. However, body expressions and postures provided sufficient information about the victim’s emotional reaction and the agent’s intention.

This task is based on the comparison of three different types of scenarios (accidental harm, intentional harm, and neutral situations). This and similar versions of the EPT have been employed in numerous behavioral and neuroimaging studies in different countries assessing clinical and non-clinical populations. The ensuing results systematically show that responses to each question are modulated by the context in which the action occurs. Specifically, empathy ratings (i.e., empathic concern, discomfort, intention to hurt, and punishment) are higher for intentional harm than for accidental harm, and ratings for these kinds of scenarios are higher than those for neutral situations

**S4. Intra-group comparisons**

Intra-group comparisons revealed that IAs judged accidental harm as less permissible than attempted harm (*t* (34) = -2.88, *p* < .01). The opposite difference was observed in controls (*t* (20) = 2.62, *p* < .05), who judged attempted harm as more permissible than accidental harm.

Supplementary Table 1. Correlation matrix (Pearson index) between EFs, ToM and moral evaluation measures in IAs.

|  | Total  IFS score | ToM | Moral judgment (accidental harm) | Intentionality comprehension (accidental harm) | Punishment  (accidental harm) |
| --- | --- | --- | --- | --- | --- |
| IFS total score | 1 | .40* | .1 | -.15 | -.28 |
| ToM | .40* | 1 | .26 | -.17 | -.55** |
| Moral judgment  (accidental harm) | .1 | .26 | 1 | .14 | -.12 |
| Intentionality comprehension (accidental harm) | -.157 | -.324 | .141 | 1 | -.37* |
| Punishment  (accidental harm) | -.28 | -.55* | -.12 | -.37 | 1 |

A single asterisk (*) indicates significant differences at *p* < .05

A double asterisk (**) indicates significant differences at *p* < .01

**Supplementary references**

Akitsuki Y., & Decety, J. (2009). Social context and perceived agency affects empathy for pain: an event-related fMRI investigation. *Neuroimage, 47*(2), 722-734. doi: 10.1016/j.neuroimage.2009.04.091

Baez S., Herrera, E., Gershanik, O., Garcia, A. M., Bocanegra, Y., Kargieman, L., Manes, F., & Ibanez, A. (2015). Impairments in negative emotion recognition and empathy for pain in Huntington's disease families. *Neuropsychologia, 68*, 158-167. doi: 10.1016/j.neuropsychologia.2015.01.012

Baez S., Herrera, E., Villarin, L., Theil, D., Gonzalez-Gadea, M. L., Gomez, P., Mosquera, M., Huepe, D., Strejilevich, S., Vigliecca, N. S., Matthaus, F., Decety, J., Manes, F., & Ibanez, A. M. (2013). Contextual social cognition impairments in schizophrenia and bipolar disorder. *PloS one, 8*(3), e57664. doi: 10.1371/journal.pone.0057664

Baez S., Manes, F., Huepe, D., Torralva, T., Fiorentino, N., Richter, F., Huepe-Artigas, D., Ferrari, J., Montanes, P., Reyes, P., Matallana, D., Vigliecca, N. S., Decety, J., & Ibanez, A. (2014). Primary empathy deficits in frontotemporal dementia. *Frontiers in aging neuroscience, 6*, 262. doi: 10.3389/fnagi.2014.00262

Baez S., Morales, J. P., Slachevsky, A., Torralva, T., Matus, C., Manes, F., & Ibanez, A. (2016). Orbitofrontal and limbic signatures of empathic concern and intentional harm in the behavioral variant frontotemporal dementia. *Cortex; a journal devoted to the study of the nervous system and behavior, 75*, 20-32. doi: 10.1016/j.cortex.2015.11.007

Baez S., Rattazzi, A., Gonzalez-Gadea, M. L., Torralva, T., Vigliecca, N. S., Decety, J., Manes, F., & Ibanez, A. (2012). Integrating intention and context: assessing social cognition in adults with Asperger syndrome. *Frontiers in human neuroscience, 6*, 302. doi: 10.3389/fnhum.2012.00302

Decety J., Michalska, K. J., & Kinzler, K. D. (2012). The contribution of emotion and cognition to moral sensitivity: a neurodevelopmental study. *Cerebral cortex, 22*(1), 209-220. doi: 10.1093/cercor/bhr111

Escobar M. J., Huepe, D., Decety, J., Sedeno, L., Messow, M. K., Baez, S., Rivera-Rei, A., Canales-Johnson, A., Morales, J. P., Gomez, D. M., Schroeder, J., Manes, F., Lopez, V., & Ibanez, A. (2014). Brain signatures of moral sensitivity in adolescents with early social deprivation. *Sci Rep, 4*, 5354. doi: 10.1038/srep05354

Gonzalez-Gadea M. L., Herrera, E., Parra, M., Gomez Mendez, P., Baez, S., Manes, F., & Ibanez, A. (2014). Emotion recognition and cognitive empathy deficits in adolescent offenders revealed by context-sensitive tasks. *Frontiers in human neuroscience, 8*, 850. doi: 10.3389/fnhum.2014.00850

Hesse E., Mikulan, E., Decety, J., Sigman, M., Garcia Mdel, C., Silva, W., Ciraolo, C., Vaucheret, E., Baglivo, F., Huepe, D., Lopez, V., Manes, F., Bekinschtein, T. A., & Ibanez, A. (2016). Early detection of intentional harm in the human amygdala. *Brain : a journal of neurology, 139*(Pt 1), 54-61. doi: 10.1093/brain/awv336

Torralva T., Roca, M., Gleichgerrcht, E., Lopez, P., & Manes, F. (2009). INECO Frontal Screening (IFS): a brief, sensitive, and specific tool to assess executive functions in dementia. *Journal of the International Neuropsychological Society : JINS, 15*(5), 777-786. doi: 10.1017/S1355617709990415
